# A 3D total-body photography research network: the Australian experiment

**DOI:** 10.1007/s00105-021-04938-7

**Published:** 2022-01-14

**Authors:** Chantal Rutjes, Joachim Torrano, H. Peter Soyer

**Affiliations:** 1 The University of Queensland Diamantina Institute, Dermatology Research Centre, The University of Queensland, 37 Kent Street, Woolloongabba, QLD 4102 Brisbane, Australia; 2 Department of Dermatology, Princess Alexandra Hospital, Brisbane, QLD Australia

## Introduction

The melanoma incidence in Australia is 12 times the global average [10], with over 15,000 Australians diagnosed annually [2]. Increasing incidence and mortality rates pose a burden to the healthcare system and quality of life of melanoma patients and their support network [6]. Supported by the Australian Cancer Research Foundation, the Australian Centre of Excellence in Melanoma Imaging and Diagnosis (ACEMID) research program aims to reduce the overarching burden of melanoma.

## This is new!

### Melanoma screening and surveillance

Specialised surveillance of individuals at a high risk of developing melanoma appears to be cost effective from preliminary studies showing it can lead to a decrease in yearly healthcare costs in Australia of more than 22 million AUD [14]. Total-body photography (TBP) is a recommended screening and surveillance method for individuals at a high risk of developing melanoma [1]. Traditional serial TBP is suggested to be more cost effective and associated with lower excision rates as well as improved prognosis compared to standard care [9]. Three-dimensional TBP (3D TBP) is one of the latest advances in melanoma screening and has the potential to overcome certain limitations of traditional TBP, as it is less time consuming and more accurate (i.e. less chances of missing naevi due to differences in camera angles and positioning of the body) [13]. Moreover, it keeps a precise record of the patient’s skin surface at various timepoints, providing a comparative record of high-quality total body images for aiding in identification of new, changing and regressing lesions [8]. Here, we present the background, aims, and status quo of the ACEMID research program that was formally launched in September 2021.

### Program background

The focus of the ACEMID research program is on early detection of melanoma and reduction of the overarching burden, morbidity, mortality and costs associated with melanoma diagnosis. A network of 15 3D TBP systems will be established, linking urban, regional and remote hospitals or research sites across Queensland, New South Wales and Victoria via an integrated telehealth network. This will yield greater clinical outcomes in terms of health expenses, time needed for skin checks and disease morbidity, especially for those living in regional and remote areas with limited access to primary healthcare and diagnostic services [4, 5]. Individuals with a melanoma history or multiple atypical moles have a 34-fold increased incidence of melanoma [3]. The project will enable early and accurate detection of melanoma in these individuals who are at an ultra-high risk for melanoma and facilitate cost-effective screening for individuals at high risk and low risk.

The 3D TBP will be conducted using the Vectra® WB360 System (Canfield Scientific Inc., Fairfield, NJ, USA), which can capture cross-polarized images of more than 95% of the patient’s skin surface to create a 3D total-body avatar of the patient [13]. This highly innovative technology was the first publicly available 3D TBP system in the world. Only a few clinical trials using 3D TBP are being conducted, such as a randomized controlled trial evaluating consumer acceptance of 3D TBP and comparing clinical and health economic outcomes of 3D TBP with standard care [12]. The 15 systems in the ACEMID research program will be used for objective and secure data collection, producing highly advanced avatars with attached sequential digital dermoscopy imaging (SSDI) to detect melanoma at an early stage. These secure databases will incorporate not only high-resolution 3D avatars and SSDI, but also data from genomic sampling and psychological/behavioural questionnaires which will develop the world’s largest, most comprehensive skin imaging database and pave the way for future melanoma research. Furthermore, it will be used to identify new naevi and other skin lesions, track and trace naevi over time, and lead to an improved understanding of naevi patterns and the way they change over time.

### Program aims

**Fig. 1:**
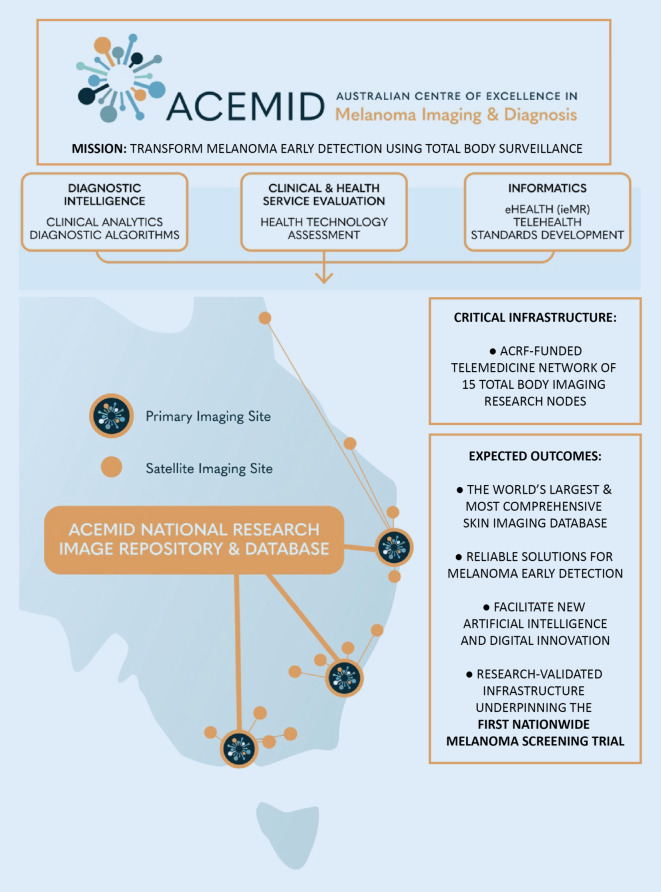
ACEMID overview. A graphic overview of the ACEMID mission, aims, critical infrastructure and expected outcomes at the commencement of the project. *ACRF* Australian Cancer Research Foundation

The project’s core infrastructure includes three connected research programs focusing on diagnostic intelligence, clinical and health service evaluation, and informatics (Fig. 1). These research programs are designed to ensure that early detection of melanoma during the 3‑year study period is achieved at a suitable level. The 3D TBP network allows for data capture and processing from multiple sites across Australia, providing the ACEMID multidisciplinary team of researchers and associated professionals with a wealth of data that cannot be collected in any other setting. Important factors for melanoma risk stratification, such as patient demographics and personal and family skin cancer history [11], will be integrated with the 3D total-body avatars.

The Diagnostic Intelligence program will develop AI models that combine the patient’s personal risk factors, including melanoma history, phenotype and genotype, and the distribution of the patient’s skin lesions within the context of the whole-body skin surface and skin lesion universe. These models will be tested until they are adequately reliable and valid in providing risk estimates at a person or lesion level that will ultimately support clinical decision-making.

The chief investigators of the Queensland research site have been researching the properties, effectiveness and workflow of the 3D TBP systems. This research includes training a new role specialising in advanced skin imaging (melanographers) and remote-access dermatologists to help the offline or remote assessment process, and assessing the health economic impact and comparing healthcare costs of 3D TBP to those of standard care. Rigorously designed questionnaires will be included for participants to determine the behavioural and psychological impact of these technologies. Regular committees and forums are also being created for engaging with academics, health departments, consumers, professional societies and other key stakeholders. The Clinical and Health Service Evaluation research program will expand this research across the Australian states of Queensland, New South Wales and Victoria, and develop quality of service assurance standards as well as economic and business models of practice, considering factors that influence implementation and the process and the consequences of implementation.

The ACEMID research program proposes a model that integrates digital health records for offline or remote assessment by a qualified healthcare professional. The abundance of imaging data collected in the project will be transmitted to centralised image storage repositories, enabling dermatologists to review images and provide a diagnosis independent of time, location or population-density parameters. The Informatics program will assess system performance and reliability of image acquisition and storage, in order to develop frameworks based on FAIR principles for data stewardship [15], design solutions for tele-dermatological imaging, reading and reporting, and establish evidence for translation to standard clinical workflows. The establishment of clinical and research repositories for these 3D images, dermoscopic images, questionnaire responses, genomic data, health records and clinical history and other associated data will facilitate further studies into development of skin lesions and the connotations of melanoma risk for individuals, as well as technological advancements such as artificial intelligence (AI).

In the years that follow the initial 3‑year study period, the ACEMID research program will be dedicated to work on two additional key implementation programs, focusing on tele-surveillance for individuals at ultra-high risk of developing melanoma, and a national screening program for high-risk melanoma patients.

### Program status quo

It is estimated that each 3D TBP system can support approximately 700 examinations per year once fully established, leading to over 30,000 digital 3D total-body avatars from all 15 systems after 3 years. This large dataset of whole-body skin and lesion images will be used to train AI models that improve diagnostic accuracy and early detection of melanoma. Improved diagnostic accuracy will have a positive effect on healthcare costs, as costs associated with diagnostic biopsies of benign lesions account for more than 70 million AUD annually [7]. In addition, implementing new tools to improve early detection of melanoma will result in the diagnosis of thinner (< 1 mm) melanomas that require less extensive and costly treatment, similarly resulting in reduced health care costs as well as improved patient quality of life and survival rates. Research findings will be translated into technical and clinical practice guidelines. The ACEMID research program project will also improve convenience and precision of naevus documentation and provide a greater proportion of the population with access to risk-stratified screening.

## Conclusion for practice

The ACEMID research program is the first of its kind in the world, representing a next-generation, precision strategy of skin imaging technology integrated within an interconnected 3D TBP telemedicine network that is poised to enable further innovation. It has the potential to change clinical decision-making by integrating AI support with patient triage and image analysis, leading to increased diagnostic accuracy by improving lesion identification and reducing appointment times and healthcare costs.

## Declarations

This article does not contain any studies with human participants or animals performed by any of the authors.
